# The House We Live in

**Published:** 1896-02

**Authors:** 


### The House We Live in.

This is the advice of the late lamented Prof. J. M. Coates: "Think deliberately of the house you live in, your body; make up your mind firmly not to abuse it; eat nothing that will hurt it; wear nothing that distorts or pains it; do not overload it with victuals or drink or work; give yourself regular and abundant sleep; keep your body warmly clad. At the first signal of danger from the thousand enemies that surround you, defend yourself. Do not take cold; guard yourself against it; if you feel the first symptoms, give yourself heroic treatment; get into a fine glow of heat by exercise; take a vigorous walk or run, then guard against a sudden attack of perspiration. This is the only body you will ever have in this world. A large share of the pleasure and pain of life will come through the use you make of it. Study deeply and dilligently the structure of it, the laws that should govern it, and the pains and penalties that will surely follow a violation of every law of life or health."—*Indiana Medical Record.*

DON'T use soap and water on the body just preceding the application of cocaine; the alkali destroys the anæsthetic action.

